# Ethical use of big data for healthy communities and a strong nation: unique challenges for the Military Health System

**DOI:** 10.1186/s12919-024-00308-y

**Published:** 2024-10-15

**Authors:** Tracey Perez Koehlmoos, Jessica Korona-Bailey, Jared Elzey, Brandeis Marshall, Lea A. Shanley

**Affiliations:** 1 Uniformed Services University of the Health Sciences, 4301 Jones Bridge Road, Bethesda, MD 20814 USA; 2 The Henry M. Jackson Foundation for the Advancement of Military Medicine Inc. 6720 Rockledge Dr, Bethesda, MD 20817 USA; 3 Nurture the Next, 600 Hill Ave, Suite 202, Nashville, TN 37210 USA; 4 DataedX Group, Smyrna, GA USA; 5 International Computer Science Institute, 2150 Shattuck Ave Suite 250, Berkley, CA 94704 USA

**Keywords:** Big Data, Ethics, Military Health System

## Abstract

Recent advances in artificial intelligence (AI) created powerful tools for research, particularly for extracting meaningful insights from extremely large data sets. These developments increase research benefits of big data and risks posed to individual privacy, forcing a re-examination of ethics in research which is of particular importance to the Military Health System. To advance discussion of research ethics in this context, the Forum on Health and National Security: Ethical Use of Big Data for Healthy Communities and a Strong Nation was held in December 2018. The workshop was designed to identify ethical questions relevant to population and health research studies using difficult to access, health-related data in the Department of Defense (DoD). Discussions explored researchers’ ethical obligations to research subjects, particularly in the areas of privacy, trust, and consent, as well as potential methods to improve researchers’ ability to collect, access, and share data while protecting privacy and potential risks to national security. These include creating risk management frameworks and data governance policies, improving education and workplace training, and increasing community involvement in research design and practice. While the workshop was conducted in 2018, the discussion of data ethics is still relevant today. The research agenda of the nation is best served by building ethics into the research ecosystem. There are substantial challenges to fully realizing this goal including commitments of time and funding to address the ethical complexities, train others to understand them, and create appropriate ethical frameworks before research begins.

## Introduction

Researchers are increasingly applying artificial intelligence (AI) to large data sets to advance health research. For example, using AI for research can include developing natural language processing models to process large datasets at a faster speed compared to human computation [1]. While this is being done with the worthy goal of improving the health of communities, it is essential to ensure that ethical considerations are woven throughout the entire process to mitigate potential negative consequences to individuals and the nation. Unfortunately, the path to doing this is not always clear. In medical research, data come from multiple sources with a number of stakeholders and undergo iterations in increasingly complex, confusing, and vulnerable systems.

The Military Health System (MHS) provides an example of the type of large datasets used for research. This system serves 9.6 million beneficiaries, approximately 80% non-active duty, and includes 1.9 million children. The beneficiary population is universally insured, able to receive care either from military facilities or in the private sector, and is nationally representative and socio-demographically diverse [2–4]. The longitudinal claims data for this population is centrally maintained in a system called the MHS Data Repository (MDR), accessed through the MHS Information Platform (MIP), and includes multiple databases for inpatient care, outpatient care, laboratory results, pharmacy data, and others [5, 6]. This positions the MHS, as one article described it, as “America’s ‘Undiscovered’ laboratory for Health Services Research” [6]. However, the specific context of the database poses significant challenges not found in other health systems.

A review of the MHS in 2014 identified “a major gap in the ability of the MHS to analyze system-wide health care information” [7]. The final report stated that “although the MHS has a wealth of data, the ability to analyze those data and use the results to guide decision making in quality and patient safety is nascent” [7]. The review highlighted “performance variability” indicating that better analytics are required to support policy, and must be aligned with training and education programs [7].

In the context of the MHS and health-related data, the use and application of AI can carry threats to national security as well as implications for individual privacy. Additionally, there are a number of ethical considerations and challenges when it comes to big data in government agencies or government-funded research institutions. Such institutions must follow regulations while private businesses have more freedom in their decision making and the ability to curate and use data. Ongoing discussions at the intersection of ethics and big data in the military health field led to a need to explore these areas more deeply and solicit diverse ideas surrounding ethics of combining big data, AI tools, and military health and performance information.

To this end, The Forum on Health and National Security: Ethical Use of Big Data for Healthy Communities and a Strong Nation was convened on 10 December 2018 at the Uniformed Services University of the Health Sciences (USUHS) in Bethesda, MD. This workshop was developed in partnership between the Health Services Research Program and the Center for the Study of Traumatic Stress of the USUHS, and the South Big Data Innovation Hub funded by the National Science Foundation. In total, 22 individuals attended the meeting from universities, federal agencies, and non-profits. The workshop was designed to identify ethical questions relevant to military health research studies using big data. Its stated objectives were to identify key ethical issues, determine mechanisms to mitigate harms, identify gaps in research systems, and identify possible solutions. The following text summarizes highlights from the day’s discussion surrounding the best ethical approaches to research with large data sets of health information. Emphasis is placed on unique challenges faced by health services researchers in the Department of Defense (DoD). While the workshop was conducted in 2018, proceedings are still relevant today.

## Ethical challenges of big data

While the world is awash in data, the term “big data” refers to data from many sources; merged with other data; from multiple time periods, data points, servers, and metadata. Big data represents actual people. Therefore, studies require consideration of data as conscientiously as one would for human subjects or whole populations. All components of big data, including collection, security, access, and analysis have ethical challenges [8]. Military health data has an extra ethical challenge. Primary health data is readily accessible for patient care, but secondary uses, such as for research that can improve health delivery requires special considerations. For example, it can be challenging to find analysts with appropriate data and security expertise who will choose to work in government rather than industry.

Ethics are especially important in military health research because scientists have a dual obligation to protect people and to protect national security [9, 10]. For example, health data of individual armed service members or their families could indicate troop deployment movements that may have national security implications. Health data is particularly sensitive as it can uncover information an individual may want to keep private. While a number of laws are in place that address privacy or security of health data, including the Health Insurance Portability and Accountability Act (HIPAA), there is still risk in using health data. For example, data could be used in ways researchers or study subjects never intended.

## Balancing use and safety of big data

A glaring issue in the DoD is the challenge of acquiring data itself. The MHS has health records for millions of service members and their families creating a rich repository for health services research yet, in order to have access to health data in the MHS a number of requirements need to be met. Some of which are acquiring necessary clearances and training of personnel. Then comes the challenge of linking together the various DoD datasets needed to answer a research study and the time getting through the approval process of Institutional Review Boards (IRBs) and various external reviews for data privacy. While these safeguards are in place to protect human subjects and national security, there must be a balance between answering high priority questions to improve the MHS and refining the system to improve the health and healthcare of approximately 9.6 million beneficiaries.

## Potential solutions

Ethics is a holistic endeavor. Big data health research requires ethical considerations in each step of the research process: from designing the research question to determining effective data collection methods to creating algorithms for analysis. Multiple ways to embed ethics into the broader research endeavor exist including: creating effective risk management frameworks and data governance policies; improving education, trust, and diversity; learning from existing systems; rethinking the approval process; and reframing the human-AI relationship as a collaboration instead of a competition.

### Risk management frameworks

The risks of using AI on health data are complex, and often unfamiliar. The creation of an ethical risk management framework that identifies problems, assesses risk, makes mitigation plans, communicates risk, seeks feedback, considers the community and reassesses risk can facilitate ethical research. While big data is diverse it is still possible to create a framework that facilitates researchers acting ethically, communicating risk, and encouraging innovation while being malleable enough to adapt to the range of projects. Risk-prevention mechanisms can be designed and incorporated enabling researchers to add resilience to a system or strengthen security features.

Despite the best intentions, however, problems are inevitable. Data cannot be “recalled” like a flawed consumer product. It can be endlessly copied or transferred and become untraceable. Ethical and effective risk management frameworks can mitigate problems and ensure consideration of ethical approaches to actions. However, following a risk management framework can create extra work. Developers or analysts may need a tangible incentive to take it on, in addition to a better awareness of the risks of not handling data ethically. A code of conduct or checklist can also nudge workers to “do the right thing.”

### Data governance

Data governance is an essential element for ethical big data research. Current data management systems have limited ability to handle today’s challenges. Ethical data governance ensures that data is findable, accessible, interoperable, and reusable. It also requires a knowledge of risk management tools and mechanisms for predicting and mitigating risks. Organizations are increasingly appointing a responsible steward to oversee this process. Ethical data governance creates guidelines for issues such as limiting data collection to only that necessary to satisfy the research question, safely sharing raw data between researchers, and restricting large data set transfers. There is a growing awareness that sharing data can increase risk. Cybersecurity is an essential piece of the ethical puzzle. Data governance plans must include proper security for data, and concerns about cloud-based services, expense, risk, and failure mitigation must also be considered.

Different research questions require different data, multiple algorithms, or separate analyses. Secure data repositories are a core part of data governance, providing researchers with different levels of access depending on their associations/needs. Such data repositories can add cost and require specialized training. Data literacy is the flip side of data accessibility, and requires tools to aid data interpretation. Tools are available or in progress to improve data interpretation among the public and to encourage researchers to consider data literacy throughout the entire research process. Data governance plans must also ensure that data is transparent and usable. Many organizations are working on improving the usability of data including the Veteran Affair (VA) and DoD through their development of new platforms.

### Education

Education across the spectrum of stakeholders, including ethical big data research for students, scientists, developers, and community members is important. Ethical use of big data should be integrated into the overall data and analytic education. Several organizations are working to create an ecosystem of responsible big data use. Industries can be encouraged to adopt and publicize their practices to establish transparency and foster trust. The California Consumer Privacy Act, modeled on the European Union’s General Data Protection Regulation (GDPR), enables individuals to decide how their data can be used, including being removed completely from a system or collection. Corporations have also developed codes of data ethics with practical applications offered to employees who work with AI and big data. Some organizations have also created free ethics curricula packets for college students and expressed a commitment to diverse hiring practices as a part of being a responsible and ethical business.

### Diversity

Diversity will improve a data ethics strategy in order to correct implicit biases in both research and leadership teams. Bias is a well-known problem in AI [11]. The example of a chatbot that quickly became crude and racist after it interacted with users on Twitter illustrates the need for broad considerations when developing AI based apps [12]. A debating bot fared better when its intake was curated instead of learned in real-time, because its developers were able to control the level of bias. It may not be feasible to curate data in every situation, as big data is sometimes used secondarily to its original use case, but in public-facing applications, ethics may require it.

Ethical discussions need teams that include a diversity of backgrounds, experience, opinions, and expertise to best tackle the complex problems. Research is enhanced when scientists seek out different opinions in order to move research forward and find solutions. To the same end, initial data governance plans would be best determined by a diverse committee of experts and stakeholders who also define the role and responsibilities of a chief data strategist for the organization The chief data strategist is a professional who will use data to drive actionable decision making.

### Community involvement

Understanding the community whose members are taking part in a research project increases the community’s level of trust in a research endeavor and promotes ethical research behavior. An effective data ethics system takes a community’s culture and perspectives into consideration throughout the research process.

In addition, participants should be able to see how their data is being used, what findings emerge from the research, and whether they will be impacted by the data use. A community advisory panel whose contributions are valued can improve the research process, flag potential abuses, and approve secondary uses of data when appropriate. However, researchers must be careful not to burden one individual with the role of representing an entire community. For example, “veterans” is a community, but within it, there are veterans of different ages, male and female veterans, and urban and rural veterans, who all have different perspectives.

### Learning from other models

Ethical data practices can be borrowed from other organizations and countries that are confronting these same issues. For example, in some countries biological repositories must adhere to strict security rules, and individuals can report data concerns to a governmental ombudsman. The separation between government and industry, and related data sharing also varies by country. Industry and academia are much more integrated in some countries. Countries also vary in their concerns about large private or commercial groups collecting private information.

The publishing industry can also offer lessons, for example, the requirement that researchers state that they obtained informed consent before their research can be published. Data enclaves as in DoD and VA also offer protections for big data, limiting who can have access and how. The data protection companies may also provide valuable information and informative examples. Security precautions in this industry are significant and they tend to encrypt data, have notification when third parties access personal data, control over personal data access, and the ability for data owners to charge for data use. This process can be made transparent, private, and gives agency and financial incentives to the data owner at a time when those options are unavailable in nearly every other sphere.

### Institutional review boards (IRBs)

IRBs include safeguards to protect subjects. However, they also have multiple shortcomings that can leave data or subjects vulnerable. While existing IRB guidelines for big data use can be helpful, most IRBs are more experienced in HIPAA compliance and may not have the data, privacy, or cybersecurity expertise that ethical big data health research requires. IRBs also often do not cover every aspect of data collection. For example, some organizations may want to own the intellectual property that is the research outcome and license it for research, and IRBs rarely handle intellectual property issues.

Some organizations use other layers of oversight in addition to, or instead of, IRBs, such as information security officers to review research proposals more quickly. Other federal agencies in particular may require cybersecurity measures and be approved by the Chief Information Officer. Unfortunately, these extra layers can delay projects and frustrate researchers. In some countries IRBs are not always mandated. Some communities in the US do not rely on IRBs to protect them but instead, set up separate, representative committees to review projects from the community’s perspective, which is also a common practice in crowdsourced or citizen science projects.

### The relationship between humans and AI

AI is often viewed as in opposition to human control but in reality, collaboration between humans and AI is the key to success. There are things that machines can do better than humans, and there are things that humans can do better than machines. In big data, AI’s fast computations can give researchers more time to interpret the results, another nuanced task where humans outperform machines [12]. AI can greatly aide and improve human performance. For example, algorithms to test for tuberculosis in x-rays performed just as well as trained radiologists and could have potential use cases when resources are constrained [13, 14]. Collaboration with AI should be encouraged where it is efficient, but not overly relied upon where it does not add value. In most cases, human creativity is needed to design an AI system, fine-tune it, and analyze the outcomes. In addition, it is humans who will know when to break the rules in order to achieve justice, and when we are merely automating inequality.

## Progress since the workshop

Since the workshop in December 2018, a number of steps have been taken by the Enterprise Intelligence and Data Solutions (EIDS) component of the Defense Healthcare Management Systems (DHMS) to streamline data access while ensuring protection of human subjects. A new virtual environment is in development that will allow access to data and tools in a centralized, virtual and secure environments. This will include standardized business rules and de-identification strategies and limit exporting data to external repositories. A long-term activity of this process includes obtaining functional support from internal Defense Health Agency (DHA) organizations and other stakeholders to sustain a virtual data environment while establishing data governance processes and standard policies for research [15].

Information from this workshop has been shared through subsequent panel sessions at several annual meetings of Association of Military Surgeons of the United States. Additional workshops, sponsored by the Center for Health Services Research (CHSR) at USUHS, have continued to train interested researchers nationwide in both the restrictions and access procedures for use of MHS datasets. Finally, the CHSR has also presented multiple webinars and in-person trainings incorporating lessons learned for the ethical management of large datasets. One example was developing an educational seminar built around the 10 Simple Rules of Big Data Management that has been featured at the Marine Corps’ Institutional Review Board and other venues [9]. Focusing separately on a national group of military and civilian researchers at all levels of seniority, and on faculty at USUHS, promotes the discussion of ethical considerations throughout the research ecosystem and provides the best chance of communication with new and developing researchers.

## Conclusion

Big data is both powerful and complex, and our understanding of how best to use, interpret, and keep safe such data are new fields of work. The application of AI to big data raises the prospect of unintended consequences, which for the DoD may include threats to national security. Therefore, ethical considerations such as the benefits to the population and healthcare system by using this data to inform program reform and planning must be part of big data research from formulating the question to how to answer the question and what to do with results. Despite the best intentions and even in the context of strong cybersecurity protections, data is vulnerable to accidental misuse, intentional misuse, unauthorized secondary uses, or application pivots that endanger privacy, civil liberties, or national security. The MHS, with its longitudinally-linked databases and strict security requirements for data access, both informs discussion for other health systems and embraces opportunities to learn from them regarding the optimal balance between data security and data access.

## Data Availability

All data is contained within the workshop report.

## Abbreviations

AI: Artificial Intelligence
CHSR: Center for Health Services Research
DHA: Defense Health Agency
DHMS: Defense Healthcare Management Systems
DoD: Department of Defense
EIDS: Enterprise Intelligence and Data Solutions
GDPR: General Data Protection Regulation
HIPAA: Health Insurance Portability and Accountability Act
IRB: Institutional Review Board
MDR: Military Health System Data Repository
MHS: Military Health System
MIP: Military Health System Information Platform
VA: Veteran’s Affairs

## References

[1] Alowais SA, Alghamdi SS, Alsuhebany N, et al. Revolutionizing healthcare: the role of artificial intelligence in clinical practice. BMC Med Educ. 2023;23(1):689. 10.1186/s12909-023-04698-z. 37740191 10.1186/s12909-023-04698-z PMC10517477

[2] Schoenfeld AJ, Kaji AH, Haider AH. Practical Guide to Surgical Data Sets: Military Health System Tricare Encounter Data. JAMA Surg. 2018;153(7):679–80. 10.1001/jamasurg.2018.0480. 29617526 10.1001/jamasurg.2018.0480

[3] Health.mil. 2022. Available from https://www.health.mil/About-MHS. Cited 2022 Sept 9.

[4] Defense Health Agency. Evaluation of the TRICARE program: fiscal year 2019 report to Congress: access, cost, and quality data through fiscal years 2018. Available from: https://www.health.mil/Reference-Center/Reports/2019/07/09/Evaluation-ofthe-TRICARE-Program-Fiscal-Year-2018-Report-to-Congress. Cited 2022 Jul 7.

[5] Rhon DI, Clewley D, Young JL, Sissel CD, Cook CE. Leveraging healthcare utilization to explore outcomes from musculoskeletal disorders: methodology for defining relevant variables from a health services data repository. BMC Med Inform Decis Mak. 2018;18:10. 29386010 10.1186/s12911-018-0588-8 PMC5793373

[6] United States Defense Health Agency. MDR, M2, ICDs Functional References and Specifications. Available from: https://www.health.mil/Military-Health-Topics/Technology/Support-Areas/MDR-M2-ICD-Functional-References-and-Specification-Documents. Cited 2022 June 6.

[7] Gimbel RW, Pangaro L, Barbour G. America’s ‘undiscovered’ laboratory for health services research. Med Care. 2010;48(8):751–6. 10.1097/MLR.0b013e3181e35be8. 20613659 10.1097/MLR.0b013e3181e35be8

[8] Department of Defense. Final Report to the Secretary: Military Health System Review. 2014. Available from: https://www.health.mil/About-MHS/Military-Medical-History/MHS-Review. Cited 2022 Jul 7.

[9] Zook M, Barocas S, Boyd D, et al. Ten simple rules for responsible big data research. PLoS Comput Biol. 2017;13(3): e1005399. 10.1371/journal.pcbi. 28358831 10.1371/journal.pcbi.1005399 PMC5373508

[10] Hosek, SD. and Straus SD, Patient Privacy, Consent, and Identity Management in Health Information Exchange: Issues for the Military Health System. Santa Monica, CA: RAND Corporation. 2013. Available from: https://www.rand.org/pubs/research_reports/RR112.html. Cited 2022. Jul 7. 10.7249/rhq-03-02-09 PMC4945174 28083296

[11] Yang Y, Lin M, Zhao H, Peng Y, Huang F, Lu Z. A survey of recent methods for addressing AI fairness and bias in biomedicine. J Biomed Inform. 2024;154: 104646. 10.1016/j.jbi.2024.104646. 38677633 10.1016/j.jbi.2024.104646 PMC11129918

[12] Benke K, Benke G. Artificial Intelligence and Big Data in Public Health. Int J Environ Res Public Health. 2018;15(12):2796. 10.3390/ijerph15122796. 30544648 10.3390/ijerph15122796 PMC6313588

[13] Qin ZZ, Ahmed S, Sarker MS, et al. Tuberculosis detection from chest x-rays for triaging in a high tuberculosis-burden setting: an evaluation of five artificial intelligence algorithms. Lancet Digit Health. 2021;3(9):e543–54. 10.1016/S2589-7500(21)00116-3. 34446265 10.1016/S2589-7500(21)00116-3

[14] Qin ZZ, Sander MS, Rai B, et al. Using artificial intelligence to read chest radiographs for tuberculosis detection: A multi-site evaluation of the diagnostic accuracy of three deep learning systems. Sci Rep. 2019;9(1):15000. 10.1038/s41598-019-51503-3. 31628424 10.1038/s41598-019-51503-3 PMC6802077

[15] Costantino R. Enterprise intelligence and data solutions. Virtual presentation for Association of Military Surgeons of the United States Annual Meeting 2022; 22-25 February, Oxon Hill.

